# Commentary: setting priorities in NCD prevention and control

**DOI:** 10.1186/s12962-018-0133-8

**Published:** 2018-11-09

**Authors:** Silvana Luciani, Anselm Hennis

**Affiliations:** 1 Department of Noncommunicable Diseases and Mental Health, Pan American Health Organization, 525 23rd St NW, Washington, DC USA; 2 Department of Noncommunicable Diseases and Mental Health, Pan American Health Organization, 525 23rd St NW, Washington, DC USA

**Keywords:** Priority setting, Noncommunicable diseases, Cost effectiveness

## Abstract

Decision making in health requires the use of sound evidence and context-specific information, guided by a priority setting methodology or framework. For noncommunicable disease (NCD) prevention and control, a decision-making methodology has been applied by the World Health Organization to delineate priorities, and options for cost-effective NCD interventions. A set of 14 interventions considered very cost-effective, affordable and feasible for implementation in various resource level settings were identified. Among them, tobacco control through taxation, bans on tobacco advertising, plain packaging, and smoke free public spaces stands out as perhaps the single most important interventions to tackle NCDs.

## Commentary

Noncommunicable diseases (NCDs) are well recognized as the leading cause of mortality worldwide and a major impediment to economic development [1–3]. As such, the Sustainable Development Goals include a target of a one-third reduction in premature mortality from NCDs by 2030. While the global health community continues to address the unachieved Millennium Development Goals, notably maternal and child mortality, and confronts escalating health emergencies and disease outbreaks, it must now scale up its response to challenges posed by NCDs. Without interventions, global NCD mortality is predicted to increase by 24% from 39 million deaths in 2015 to 52 million by 2030 [4]. Addressing NCDs will require policy and health service interventions that can drastically reduce risk factor prevalence, and prevent and control NCDs, with actions not only in the health sector, but involving all sectors of government and society. Feasible and sustainable evidence-based prevention and control approaches are needed, particularly in settings with less than robust health systems, and in the face of powerful tobacco industry efforts which hamper NCD prevention efforts. Key issues then are, what are the most effective strategies to address NCDs, what actions should be prioritized, and where should limited health resources be focused to have the greatest impact, given the numerous competing health issues?

Such decision making, which is sound, efficient, and results-focused, can only be guided by utilizing a priority setting methodology or framework, several of which have been previously described [5–10]. To aid priority setting specifically for NCDs, WHO has recently applied a decision-making methodology that delineates priorities for NCD prevention and control, and provides a menu of cost-effective NCD interventions [11]. Using the WHO-CHOICE economic methodology, the cost-effectiveness ratio of evidence-based NCD interventions was evaluated by WHO, along with the expected population health impact and cost per year of implementation. Beyond the economic parameters, implementation considerations were also evaluated and included qualitative analysis to consider feasibility, equity, health system considerations, and acceptability, among others. The intervention options were derived using a ‘’bottom up approach’’, through several global key stakeholder consultations across numerous countries in the World in which governmental and non-governmental organizations discussed results of the economic evaluations and provided input on the proposed NCD interventions. These results led to an extensive list of 81 recommendations for NCD interventions, with the priorities identified as 14 interventions considered very cost-effective, affordable and feasible for implementation. Among them, tobacco control through taxation, bans on tobacco advertising, plain packaging, and smoke free public spaces stands out as perhaps the single most important interventions to tackle NCDs. Policy interventions to reduce harmful use of alcohol, such as taxation on alcoholic beverages, and restrictions on alcohol advertising; as well as interventions for salt reduction and taxation on sugar sweetened beverages are also among the cost-effective NCD interventions.

In May 2017, the 70th World Health Assembly—the ultimate decision making authority in health—endorsed these priorities for NCD interventions, known as Appendix 3 of the WHO Global NCD Action Plan 2013–2020. While Appendix 3 is a useful tool to guide decision making in health for NCDs, it will not replace the need for priority setting methodologies to be applied at the national level so that the specific context, economic parameters, feasibility of implementation can be analyzed to determine national level priorities for NCD prevention and control.

## Conclusions

Because of the sheer magnitude of the burden and impact of NCDs in all countries, it is urgent and important to make NCD prevention and control a health priority. However, making decisions on which public health interventions to prioritize, among the vast range of interventions needed to improve NCD prevention and control efforts is a challenge. The WHO NCD cost-effective interventions, which was developed using a priority setting methodology, provides a sound, technical list of priority interventions that are feasible and affordable to implement in various resource-level settings.
